# Perioperative immunotherapy in resectable HNSCC: biological rationale to practical multidisciplinary implementation

**DOI:** 10.3389/fonc.2026.1799437

**Published:** 2026-05-15

**Authors:** Michel Bila, Vincent Vander Poorten, Jeroen Meulemans, Wouter Huvenne, Wouter De Vos, Robin Willaert, Joke De Ceulaer, Paul M. Clement

**Affiliations:** 1 Dept. of Oral and Maxillofacial surgery, Head and Neck surgery, University Hospital Antwerp, Antwerp, Belgium; 2 Dept. of Translational Neurosciences, University of Antwerp, Antwerp, Belgium; 3 Vlaamse Werkgroep Hoofd-HalsTumoren (VWHHT), Gent, Belgium; 4 Laboratory of Experimental Oncology (LEO), Department of Oncology, KU Leuven, Leuven, Belgium; 5 Otorhinolaryngology - Head and Neck Surgery, Leuven Cancer Institute, University Hospitals Leuven, Leuven, Belgium; 6 Department of Oncology, section Head and Neck Oncology, KU Leuven (Katholieke Universiteit Leuven), Leuven, Belgium; 7 Department of Head and Neck Surgery, Ghent University Hospital, Ghent, Belgium; 8 Dept. of Oral and Maxillofacial Surgery, University Hospital Leuven, Leuven, Belgium; 9 Oral and Maxillofacial Surgery – Imaging and Pathology (OMFS-IMPATH) Research Group, Department of Imaging and Pathology, Faculty of Medicine KU Leuven (Katholieke Universiteit Leuven), Leuven, Belgium; 10 Dept. of Oral and Maxillofacial Surgery, AZ Sint-Jan Brugge-Oostende (General Hospital Sint-Jan Brugge-Oostende), Bruges, Belgium; 11 Department of General Medical Oncology, Universitair Ziekenhuis (UZ) Leuven, Leuven, Belgium

**Keywords:** head and neck squamous cell carcinoma, multidisciplinary care, neoadjuvant pembrolizumab, pathologic response, PD-1 blockade, perioperative immunotherapy, surgery, tumor−draining lymph nodes

## Abstract

**Background:**

Perioperative immune checkpoint inhibition (ICI) has moved from investigational window studies to phase III supported strategies for resectable, locally advanced head and neck squamous cell carcinoma (HNSCC), with direct consequences for surgical timing, neck management, reconstruction and pathology workflows.

**Methods:**

We performed a narrative review of phase III perioperative trials and key neoadjuvant studies in resectable HNSCC, focusing on outcomes and practical questions most relevant to surgeons and multidisciplinary teams (MDTs): pathway timing and attrition, operability, perioperative safety and feasibility of standardized response assessment.

**Results:**

Phase III evidence supports two complementary perioperative approaches: (i) neoadjuvant PD-1 priming followed by surgery and risk−adapted postoperative radiotherapy/chemoradiotherapy with continued PD-1 blockade, improving event−free survival compared with standard care; and (ii) postoperative nivolumab added to adjuvant chemoradiotherapy in patients with pathological high−risk features, improving disease−free survival. Across neoadjuvant programs, short preoperative ICI exposure was generally feasible without compromising resectability but requires protected timelines to avoid delays to curative treatment. Key surgical considerations include management of the tumor−draining lymph nodes, anticipation of immune−related adverse events affecting wound healing and rehabilitation, and coordination of treatment with systemic therapy. Pathologic response, preferably reported as percent residual viable tumor separately in the primary tumor bed and nodal compartments, is currently the most widely used endpoint and a prerequisite for response−adapted de−escalation or escalation studies.

**Conclusion:**

Perioperative ICI turns curative−intent HNSCC care into a tightly timed program. Successful implementation will depend on standardized pathology and radiology workflows, MDT−owned scheduling and prospective registries to define optimal sequencing and salvage after perioperative PD-1 exposure.

## 1 Introduction

### 1.1 The phase III shift

As of June 2025, the role of immunotherapy in resectable head and neck squamous cell carcinoma (HNSCC) shifted from an investigational to a regulatory standard. The FDA and subsequent EMA approval of perioperative pembrolizumab for locally advanced (LA) HNSCC with a PD-L1 Combined Positive Score (CPS) of 1 or more, administered as neoadjuvant priming followed by surgery and postoperative radiotherapy/chemoradiotherapy, fundamentally redefines the standard of care (1). This is not an incremental optimization but rather a change in how curative-intent care should be organized.

This narrative review examines perioperative immunotherapy in resectable HNSCC through a multidisciplinary lens, with particular emphasis on the biologic rationale for timing, the implications of phase III trials for pathway design, perioperative safety and reconstruction, and practical response assessment on pathology. The aim is to translate rapidly evolving data into concrete MDT decisions rather than to catalogue every investigational combination.

### 1.2 The perioperative window as an organizational problem

Historically, surgical efficiency was defined by the velocity from diagnosis to incision. With perioperative immunotherapy, immediate surgery without multidisciplinary synchronization becomes a liability. Because eligibility for the approved KEYNOTE-689 regimen relies on PD-L1 status, the presence of macroscopic disease for immune priming and patient specific factors, the decision to operate immediately and permanently closes the neoadjuvant window. Consequently, perioperative immunotherapy should be treated as a system re-engineering problem with a front-loaded multidisciplinary team (MDT) necessity instead of a drug selection problem. Diagnostic biopsy adequacy for CPS scoring, performance status optimization, prehabilitation and multidisciplinary dialogue must now occur synchronously at first presentation. Delays in the MDT pathway do not just postpone treatment, they alter the biological potential of the curative strategy.

Early neoadjuvant “window-of-opportunity” studies showed feasibility in trial-selected patients, but also revealed how fragile the sequence was outside protocolized care (2–8) Systematic syntheses reinforce that feasibility signals are strongest when preoperative intervals are short and scheduling is tightly coordinated, which is exactly what is most difficult to reproduce in real-world pathways. Pathway design starts with a simple rule: once neoadjuvant immune checkpoint inhibition (ICI) is initiated, surgery should not be pushed back for logistics or a desire to see more response. If the timeline must change, it should preferentially be brought forward to protect operability. Postponement should be reserved for clear patient−safety reasons (e.g., toxicity) and not pursued electively outside ethics committee approved prospective protocols.

A practical implementation model is to plan backward from the intended surgery and radiotherapy start date, the neoadjuvant ICI window then becomes a protected interval. Potential candidates for perioperative immunotherapy will ideally be identified at the first surgical consultation, not after completion of staging and MDT discussion. If neoadjuvant therapy is considered only after full staging, valuable time is lost while awaiting additional biomarker results (e.g., CPS) or repeat scheduling. Surgical pathways in head and neck oncology are typically organized around tightly planned operating room slots shortly after MDT review. At the first clinic visit, surgeons can usually assess the probability of eligibility based on tumor stage, general condition (absence of severe frailty), absence of immediate airway or vital threat and patient willingness to comply with systemic therapy. When this probability is high, the pathway should be organized immediately toward a perioperative strategy.

The most critical shift however is conceptual, not logistical. In the era of immunotherapy, surgery must be reconceptualized from a mechanical intervention to a biological event. Classically, the surgeon’s role was viewed as physically removing the tumor burden to achieve R0 status with acceptable quality of life (QOL). In the context of perioperative immunotherapy, surgery is also an active immunological modulator. It abruptly removes the source of antigen (the primary tumor) and the site of priming (the draining lymph nodes) and induces a profound systemic inflammatory response. This is a massive biological reset switch that can consolidate the immune memory generated by neoadjuvant therapy.

### 1.3 Pathology and biomarker discipline as surgical priorities

The introduction of perioperative immunotherapy shifts key decisions upstream in the treatment pathway, increasing reliance on pathology, biomarker assessment and pretreatment tissue adequacy. For surgeons, this has practical implications for how diagnostic samples are obtained and documented, as these factors now condition treatment eligibility and response assessment rather than serving solely as postoperative confirmation.

Regulatory approvals anchors on PD−L1 CPS thresholds. Diagnostic sampling must be planned to support CPS testing aside from Human Papilloma Virus (HPV) status. A purely cytology-based pathway does not allow a proper analysis. While cell blocks could show PD-L1 expression on tumor cells, thus indirectly inferring a CPS of 1 or higher, such approach would underestimate the true CPS rate.

HPV status is another biologic stratifier with practical consequences. HPV-positive oropharyngeal cancers often have a more inflamed baseline microenvironment and have shown higher rates of pathologic regression signals in neoadjuvant PD-1 trials compared with HPV-negative tumors (9). Yet HPV positivity does not guarantee deep response, and critically, HPV-positive disease already has favorable outcomes with standard therapy. This makes HPV-positive cohorts attractive for de−escalation concepts, but it also raises the bar for evidence: any response−adapted reduction of RT or surgery must be validated with long−term control data. On the other hand, HPV-negative disease represents a distinct biological entity characterized by genomic instability and intrinsic resistance to standard multimodality therapy. KEYNOTE-689 mainly included HPV-negative tumors, underscoring that practice changing perioperative evidence currently applies most directly to HPV unrelated disease.

Surgeons also need to anticipate that response assessment will increasingly depend on pretreatment tissue and imaging. Marking biopsy sites, documenting the extent of ulceration or infection, and sharing baseline imaging with pathology can prevent misclassification of biopsy−related necrosis or inflammation as therapy−related regression. These are small operational details, but they determine whether pathology assessments become credible enough to support future response−adapted strategies.

### 1.4 The tumor-draining lymph nodes

The strongest biologic argument for neoadjuvant ICI is not to “shrink the tumor before surgery”, it is immune priming in the presence of intact tumor antigen and an intact lymphatic network. Surgical treatment generally comprises the removal of the primary tumor and the tumor-draining lymph node (TDLN) basin. The neoadjuvant rationale rests on the principle that immunotherapy delivered while the tumor and its lymphatic drainage are intact can prime a broader and more effective systemic immune response.

The key supporting evidence is mechanistic and cross-tumor. In preclinical models, neoadjuvant immunotherapy outperformed adjuvant delivery for eradicating metastatic disease, linked to expansion and systemic dissemination of tumor-specific T cells (10). In syngeneic oral cavity carcinoma models, neoadjuvant PD-1 blockade reshaped the hierarchy of tumor-antigen responses and improved outcomes compared to adjuvant strategies (11). In HNSCC-specific models, preservation of lymphatic drainage during immune priming was particularly important. Disruption or ablation of the TDLNs reduced response to ICIs, while delaying nodal clearance significantly improved outcomes (12).

These observations align with human translational data indicating that TDLNs are not merely sites of occult spread but function as an immunological priming hub, enriched for naïve and early memory T-cell states that can be recruited into the tumor and differentiate into effector populations following checkpoint modulation (13). The practical implication is that removing the draining basin too early may truncate the pool of recruitable clones. Importantly, this does not imply that effective immune priming is impossible in the absence of intact TDLNs. Durable clinical benefit from PD-1 blockade in recurrent/metastatic (R/M) HNSCC demonstrates that antitumor immunity can be generated despite prior surgery, radiotherapy and disruption of regional lymphatic architecture (14, 15). Similarly, the NIVOPOST-OP trial shows that postoperative initiation of immune checkpoint blockade can still improve disease-free survival in patients with high-risk pathology (16).

However, these observations should be interpreted as evidence of possibility, not equivalence. The preclinical, translational and perioperative data collectively suggest that, in resectable HNSCC, intact TDLN–tumor connectivity is more likely to support efficient clonal priming, expansion and trafficking during short neoadjuvant intervals.

Collectively, these findings indicate that the timing and extent of neck intervention have become biologically consequential in the context of neoadjuvant immunotherapy. It does not challenge the oncologic importance of neck treatment but reframes the neck as both a site of disease control and a transient immunologic compartment. As a result, perioperative ICI raises MDT-relevant considerations regarding the extent of neck dissection, the role of sentinel node biopsy and the impact of elective nodal irradiation on immune priming.

### 1.5 Scheduling the neoadjuvant ICI

From a surgical and pathway perspective, the duration of the preoperative interval is a critical variable. Extended neoadjuvant windows increase exposure to interval tumor progression, patient anxiety and immune-related adverse events, all of which can jeopardize operability and erode real-world feasibility. Early window-of-opportunity studies demonstrate that immune activation can occur rapidly after PD-1 blockade and that definitive surgery can proceed safely after short neoadjuvant exposure in protocolized settings (2, 8, 17). However, the planned preoperative interval varied widely across early trials, ranging from short windows of 1–3 weeks to longer schedules extending over several weeks (2, 3, 5, 6, 9, 18).

KEYNOTE-689 mandated two 200-mg doses of pembrolizumab spaced 3 weeks apart, committing patients to a rigid 6-week window. However, pharmacokinetic modeling suggests that a single 400-mg dose provides bioequivalent exposure (19). Switching to a high-dose, single-fraction strategy would reduce the logistical burden. Mechanistically, this is plausible as well. PD-1 receptor saturation is achieved immediately upon infusion and maintained for months, meaning repeat dosing is not required to sustain target inhibition during the pre-surgical window (4, 18, 20). However, event-free survival (EFS) benefit has not been demonstrated for such a schedule and the minimal duration of neoadjuvant exposure for effective immune priming remains unknown.

While Phase II studies confirm that immune priming can occur within 3–4 weeks, this timeframe is often insufficient for the immune system to accomplish the extensive tumor clearance required for a MPR (2, 4, 18). This temporal lag creates a strategic hurdle for future response–adapted trials using pathology outcomes. Consequently, if short-course priming is adopted to improve surgical feasibility, trials may need to pivot away from standard pathology toward faster-acting biomarkers to accurately identify patients eligible for adjuvant modulation. Conversely, longer neoadjuvant intervals may increase the likelihood of measurable pathological regression, but they also introduce a higher risk of interval progression, loss of operability and attrition from the curative pathway.

Experience from melanoma and hepatocellular carcinoma warns that schedule and duration significantly influence efficacy and that perioperative immunotherapy success is context-dependent (21, 22). For resectable HNSCC, the central question is what degree of immune priming is biologically sufficient, surgically tolerable and operationally implementable within a curative pathway. Key messages from this section are summarized in **Box 1**.

Box 1 **Key messages.**

- Perioperative immunotherapy is not simply another drug added to standard surgery, it changes the organization of curative care.
- The biologic logic of neoadjuvant PD-1 depends on intact tumor antigen, preserved TDLNs and disciplined control of the preoperative timeline.
- Pretreatment tissue adequacy, CPS testing and baseline radiology and pathology now shape both eligibility and downstream response assessment.

## 2 Evidence base

### 2.1 From R/M HNSCC to curative intent

Perioperative immunotherapy in resectable HNSCC is not simply an advancement of the same drug earlier in the treatment. It is a selective translation of what worked in R/M HNSCC, combined with a recognition of what failed when ICIs were inserted into locally advanced pathways without attention to timing, patient selection or biology.

In R/M HNSCC, PD-1 blockade changed the standard of care by delivering durable benefit in a subset of patients, with improved tolerability compared with historical cytotoxic treatment. CheckMate 141 and KEYNOTE-040 established nivolumab and pembrolizumab as a survival-improving option after platinum failure (15, 23). KEYNOTE-048 then redefined first-line therapy, replacing the EXTREME regimen with PD-1–based strategies (14, 24). The key concepts from the R/M experience were that ICIs work, that achieved responses are durable, that benefit is heterogeneous (with increasing probability with higher PD-L1 scoring) and that early progression on PD-1 monotherapy is a clinically relevant phenomenon that can be mitigated by combining ICIs with chemotherapy, although the long-term survival is not substantially different (14, 15, 23, 24).

What failed was the idea that adding an ICI anywhere into definitive multimodality therapy would reliably improve cure rate. Two large definitive chemoradiotherapy (CRT) integration programs illustrate this clearly. JAVELIN Head and Neck 100 did not improve outcomes when avelumab was added to standard CRT, and KEYNOTE-412 failed to demonstrate a statistically significant EFS benefit when pembrolizumab was added to definitive CRT in an unselected population (25–27). With longer follow-up, KEYNOTE-412 showed a separation of EFS curves, but this did not translate into a statistically significant OS benefit, and the trial remained negative by its primary efficacy criteria. The negative signal became even more difficult to ignore after IMvoke010: maintenance atezolizumab following definitive multimodal therapy for high-risk LASCCHN did not improve event-free or overall outcomes versus placebo (28). These negative results force a confrontation with a critical reality: perioperative benefit is not a class effect of PD-1 blockade, nor is it achieved by simply adding the drug at any random point in the timeline. The failure of adjuvant and concurrent maintenance strategies suggests that timing, patient selection and interaction with regional immune architecture likely matter for the eradication of microscopic residual disease.

### 2.2 Tumor ecosystem and microbiome as modifiers of ICI effect

Beyond PD-L1 and conventional immune infiltrate descriptors, emerging data suggest that the tumor ecosystem, including intratumoral bacteria and myeloid-dominant inflammation, may modify the benefit of checkpoint blockade in HNSCC. Analyses of the phase III JAVELIN Head and Neck 100 dataset linked differential outcomes to distinct ecosystem states, with poorer outcomes on ICI in tumors characterized by neutrophil/myeloid activation and intratumoral bacterial signatures (29). These observations remain hypothesis-generating, but they advocate that ICI efficacy may be sensitive to exposures that alter host–tumor microbial and immune conditions.

Mechanistic work further supports plausibility by linking higher intratumoral bacterial burden to neutrophil accumulation, T-cell depletion and reduced sensitivity to ICIs. In parallel, retrospective R/M datasets associate antibiotics and proton pump inhibitors with attenuated ICI outcomes (30, 31). For surgeons, the suggested strategy forward is disciplined documentation of perioperative medications and exposures that can plausibly influence immune biology (e.g., antibiotics, PPIs, corticosteroids and their timing/dose).

### 2.3 KEYNOTE-689

Perioperative immunotherapy in resectable HNSCC is now substantiated by two phase III trials that define two different strategies rather than equivalent options. KEYNOTE-689 represents a priming approach that starts before resection, while NIVOPOST-OP embodies an intensification approach applied after high-risk pathology is proven.

KEYNOTE-689 establishes the first positive phase III evidence for perioperative PD-1 blockade in resectable LA HNSCC. This trial used a sandwich design: neoadjuvant pembrolizumab (200 mg every 3 weeks for two cycles) followed by surgery and subsequently adjuvant pembrolizumab concurrent with postoperative radiotherapy (with or without cisplatin), continuing as maintenance for 15 cycles. This regimen achieved a statistically significant EFS improvement. Again, the magnitude of benefit was greater in PD-L1–enriched subgroups, consistent with R/M experience (HR 0.66 in CPS ≥10).

While the trial met its primary EFS endpoint, the delivery metrics reveal a critical lesson. The authors report that 88% of patients successfully underwent definitive surgery and only 75% of randomized patients initiated the adjuvant phase. This 25% attrition rate within a controlled trial setting represents an inherent operational vulnerability of multimodal HNSCC treatment. In fact, these figures mirror real-world data. Analyses of the US National Cancer Database consistently show that 15–30% of eligible patients fail to start or complete guideline-adherent postoperative radiation due to surgical complications, prolonged recovery or patient refusal (32–34). The KEYNOTE-689 protocol simply unmasks this existing reality.

Real-world evidence suggests that durable disease control from ICIs does not require prolonged exposure, as immune memory can sustain remission after treatment ends. Studies show that many long-term responders maintained stability despite early discontinuation, sometimes after minimal exposure (35–37). While immune-related adverse events (irAEs) are a common cause for stopping therapy, they actually served as the strongest predictor of superior overall survival (OS) (HR = 0.38) in R/M HNSCC. Consequently, early treatment cessation, especially when triggered by irAEs, does not appear to compromise long-term efficacy.

While these data cannot be directly extrapolated to the curative perioperative setting, they support the biological plausibility that effective immune priming may occur early and that treatment duration alone is an imperfect surrogate for durable benefit. These studies challenge the need for a full 17-cycle adjuvant course as prescribed in KEYNOTE-689 as mandatory for cure. If the immunological training is achieved by the neoadjuvant priming, then the inability to complete the full adjuvant course may be less clinically detrimental than traditional chemotherapy or radiotherapy interruptions (12).

Nevertheless, extrapolating data from the R/M setting to the curative perioperative setting requires caution. In R/M disease, long-term survival is often measured in years of disease control, whereas the goal in the curative setting is permanent eradication. It remains unknown whether the two neoadjuvant doses administered in KEYNOTE-689 constitute the minimal effective dose required to sterilize microscopic disease in the absence of the adjuvant tail.

### 2.4 NIVOPOST-OP

NIVOPOST-OP defines perioperative immunotherapy at the opposite end of the pathway: postoperative escalation for patients who declare high-risk biology on surgical pathology. In this randomized phase III trial, resected LA SCCHN patients with at least one high-risk feature (extranodal extension (ENE), microscopically positive margins, ≥4 involved cervical nodes without ENE or perineural invasion) received postoperative CRT with or without nivolumab (16). Nivolumab was delivered in a single pre-CRT lead-in dose, followed by three concomitant doses during CRT, and six maintenance doses thereafter. Disease-free survival (DFS) improved (HR 0.76; p=0.034), independent of PD-L1 expression in the trial report. Toxicity increased with treatment-related grade 4 adverse events reported in 10% vs 5% and treatment-related deaths occurring in two patients in each group.

NIVOPOST-OP should be interpreted against the broader record of attempts to combine ICIs with curative-intent chemoradiotherapy. JAVELIN Head and Neck 100 (avelumab plus definitive CRT followed by maintenance) used a single lead-in dose given 7 days before CRT and did not improve progression-free survival (PFS) (26). KEYNOTE-412 similarly did not demonstrate a significant improvement in EFS at the prespecified threshold despite delivering pembrolizumab q3w for up to 17 doses, including one dose before CRT, two during CRT and prolonged maintenance (25). In the postoperative frame, IMvoke010 (maintenance atezolizumab after multimodal definitive therapy in high-risk LA HNSCC) was also negative for EFS (28). These negative trials argue against simplistic assumptions that ICI scales into curative CRT by default. They also highlight that NIVOPOST-OP differs in ways that plausibly matter, without proving which element is causal.

One biologically plausible differentiator is sequencing. NIVOPOST-OP included a genuine pre-CRT lead-in phase (one full nivolumab cycle 2w before CRT), whereas JAVELIN HN100’s and KEYNOTE-412’s administered only a short lead-in of 7 days before the start of concurrent chemoradiotherapy. The clinical relevance of that difference remains uncertain, but it intersects with a well-described antagonist in this setting: treatment-related lymphopenia. In HNSCC specifically, Campian et al. reported that during concurrent CRT, median total lymphocyte count fell by 73%, with approximately 60% of patients meeting criteria for severe lymphopenia (38). Modelling studies of fractionated radiotherapy suggest that over a conventional treatment, a very large proportion of circulating blood/lymphocytes may receive low but biologically meaningful radiation doses (e.g., after 30 fractions, 99% of circulating blood receiving ≥0.5 Gy), supporting the concept that irradiation of circulating immune cells can contribute to lymphopenia (39). This may suggests that initiating PD-1 blockade well before CRT-associated lymphopenia may improve the odds that an immune response is initiated and sustained.

A second differentiator is risk selection. NIVOPOST-OP restricts therapy escalation to patients with high-risk pathological features, increasing event rates and improving the chance of detecting a signal, while also targeting a cohort where residual microscopic disease and immune escape may be more clinically relevant. This is a more coherent use of intensification than treating broad unselected definitive CRT populations, where heterogeneity in biology and baseline risk can dilute benefit.

In NIVOPOST-OP, intensification came with a clinically meaningful toxicity: grade 4 irAEs occurred more often in the nivolumab arm (10% vs 5%), and treatment-related deaths were reported in both groups. In the postoperative curative setting, this is not a cosmetic trade-off. Any grade 3–4 treatment-related toxicity, whether driven by CRT or irAEs, can compromise regimen deliverability by interrupting chemoradiotherapy or reducing adherence to maintenance ICI. Excess grade 4 events were concentrated in the early period (≤100 days), underscoring that clinically meaningful toxicity can occur on the same timeline as CRT delivery. At the same time, the trial raises a legitimate question: Can the immune activation that generates clinical benefit also manifest as immune-related toxicity as in R/M HNSCC? But it also motivates to determine whether any toxicity–benefit coupling exists, and if so, whether it is driven by specific irAE phenotypes, timing or immune signatures.

### 2.5 Integrating KEYNOTE-689 and NIVOPOST-OP

These two trials create a strategic conflict that the MDT must face explicitly. If a patient presents with resectable LAHNSCC, KEYNOTE-689 argues for a neoadjuvant start to exploit intact tumor antigen and TDLN biology, NIVOPOST-OP argues for reserving intensification for those with confirmed high-risk pathology after resection. However, these trials should not be positioned as interchangeable options, they modify the disease trajectory in fundamentally different ways.

In KEYNOTE-689, the EFS benefit is driven mainly by a reduction in distant metastatic disease. This confirms the priming hypothesis in intact TDLNs to generate a systemic circulating T-cell repertoire capable of seeking and destroying micrometastases. Conversely, the benefit in NIVOPOST-OP was driven primarily by improved LRC. In the adjuvant setting, the TDLNs have been surgically removed. Consequently, the immunotherapy likely acts locally to sterilize the surgical bed but fails to mount systemic patrol to suppress distant recurrence.

A practical MDT synthesis is to treat them as sequential strategies rather than parallel options:

- Plan A (Priming): Neoadjuvant PD-1 followed by surgery when deliverable without jeopardizing timeliness or operability (KEYNOTE-689). This is the biological ideal, utilizing the TDLNs to mount an immune response before resection.
- Plan B (pathology-guided escalation): Upfront surgery when neoadjuvant delivery is not feasible (e.g., airway threat) or unsafe. Intensification (NIVOPOST-OP) is then deployed only if high-risk pathology (ENE+) justifies the toxicity and resource burden.

This framing clarifies the surgeon’s accountability. If the MDT chooses Plan A, the surgeon is responsible for protecting the neoadjuvant interval. If the MDT chooses Plan B, the pathologist is responsible for the assessment of high-risk features that trigger escalation (**Table 1**).

**Table 1:** Comparison of KEYNOTE-689 and NIVOPOST-OP perioperative immunotherapy strategies in resectable locally advanced HNSCC.

Feature	KEYNOTE-689	NIVOPOST-OP
Primary Strategy	Neoadjuvant Priming: Exploit intact tumor and draining lymph nodes prior to surgical disruption.	Postoperative Escalation: Pathology-guided intensification reserved for proven high-risk disease.
Target Population	Resectable, locally advanced HNSCC.	Resected LA-HNSCC with high-risk pathological feature.
Treatment Timing	Sandwich: Neoadjuvant (2 cycles) > Surgery > Adjuvant (concurrent with RT/CRT) > Maintenance.	Postoperative: Lead-in dose (pre-CRT) > Concomitant (during CRT) > Maintenance.
Immunological Rationale	Intact TDLNs facilitate robust, systemic T-cell expansion before resection.	Pre-CRT lead-in PD-1 blockade to establish immunity before radiation-induced lymphopenia.
Efficacy Driver	EFS benefit driven primarily by a reduction in distant metastatic disease.	DFS benefit driven primarily by improved LRC.
Biomarker Dynamics	Magnitude of benefit is enriched by PD-L1 positivity	Benefit reported as independent of baseline PD-L1 expression.
Key Vulnerabilities	Deliverability: High attrition rate (~25%) between surgical resection and initiation of the adjuvant phase.	Toxicity: Increased incidence of Grade 4 immune-related adverse events during the vulnerable CRT window.
Proposed MDT Integration	Plan A: The biological ideal. Utilized when neoadjuvant window is safe and does not threaten operability.	Plan B: The pragmatic fallback. Utilized when upfront surgery is mandatory (e.g., airway threat, rapid progression, etc.); escalated only if pathology dictates.

The two phase III trials represent complementary approaches: neoadjuvant priming (KEYNOTE-689) versus postoperative pathology-guided escalation (NIVOPOST-OP).

## 3 Neoadjuvant window studies

Before KEYNOTE-689, neoadjuvant immunotherapy in resectable HNSCC was largely developed through window-of-opportunity phase I/II studies. In 2026 these trials remain essential because they reveal what perioperative ICIs can deliver reliably, what they cannot yet deliver and which surgical outcomes the field still fails to measure.

### 3.1 Feasibility is real but requires protected timelines

Across early PD-1/PD−L1 window−of−opportunity studies, short neoadjuvant exposure rarely prevented surgery in trial−selected populations, and major unplanned surgical delays were uncommon (2, 4–6, 9, 18, 40). However, feasibility in trials reflects an engineered environment: expedited imaging and pathology, protected operating room access and eligibility criteria that exclude many patients most vulnerable to pathway failure (frailty, unstable comorbidity, complex airway, infection, rapidly progressive tumors).

In routine practice, the feasibility of neoadjuvant ICI therefore depends on the reliability of diagnostic and referral pathways. This vulnerability is not hypothetical. Population-level analyses consistently show that treatment delays remain common and can worsen when systems are under pressure (41). In the curative management of locally advanced HNSCC, the most actionable metrics remain time to surgery and time from surgery to postoperative radiotherapy. Because delayed PORT is associated with worse OS, perioperative regimens must be implemented without compromising the established benchmark of initiating radiotherapy within 6 weeks (42). Also, when the neoadjuvant window is missed, the patients do not simply “miss a drug”, they lose a biological opportunity to prime systemic immunity.

### 3.2 Early efficacy signals were reproducible, but depth and consistency were limited with monotherapy

Window studies consistently show biological activity and measurable pathologic regression in a subset of patients, but they also demonstrate the limits of single-agent PD-1 therapy in unselected resectable HNSCC. In Uppaluri et al., pathologic tumor response categories were observed despite the short preoperative interval, yet pathologic complete response was rare (4). In Wise-Draper et al., pathologic response occurred in 39% overall and major pathologic response (MPR) in 7%, with higher response rates in intermediate-risk compared with high-risk postoperative strata (18, 43). Importantly, pathologic response correlated with improved DFS, supporting pathologic response as a potential biological intermediate endpoint, while simultaneously highlighting that most patients do not achieve deep pTR with PD-1 alone.

Pathologic treatment response (pTR) is an attractive intermediate endpoint in neoadjuvant immunotherapy because it is available at resection long before survival endpoints mature. Cross-tumor data (e.g., melanoma and resectable NSCLC) support its potential prognostic relevance (44, 45), but in mucosal HNSCC pTR remains methodologically fragile. A recent systematic review and meta-analysis reported associations between partial/major pathologic response and DFS, but not OS, and highlighted substantial heterogeneity in definitions, compartments scored (primary vs nodes) and sampling (46). Discordant responses between the primary tumor and nodal metastases are also reported, reinforcing that composite response metrics can mask clinically relevant compartment-specific biology. These limitations underscore the need for harmonized, compartment-specific response assessment.

Several window trials intentionally used short neoadjuvant intervals to avoid compromising operability and were therefore not designed to maximize macroscopic tumor regression. Although immune priming and clonal expansion may occur early, histologic tumor clearance can lag. The absence of MPR or pCR after a 1–3-week window should therefore not be interpreted as biological inefficacy, but may reflect sampling early in the response trajectory.

Evidence from HNSCC neoadjuvant studies supports this kinetic separation. Schoenfeld et al. noted that additional cycles or a longer interval before surgery would likely have produced greater tumor regression, acknowledging time dependence while recognizing that their trial was not designed to test it (2). In contrast, a response-adaptive surgical timing study used early imaging to determine whether neoadjuvant immunotherapy should be extended before surgery, demonstrating improved pathological responses when treatment was prolonged in early imaging responders (47).

However, time alone is insufficient to guarantee deep pathologic responses in HNSCC. CIAO executed an 8-week window in largely HPV-associated disease. But despite signals of immune activation and some deep responses in nodal disease, strict MPR remained uncommon (5). Similarly, KEYNOTE-689 still reported low pCR and modest MPR rates despite a longer neoadjuvant interval with two cycles over 6 weeks and clear EFS benefit (1). The correct inference is that pathological response is endpoint-sensitive as well as time-sensitive, and it may lag behind the immune priming that actually drives systemic benefit. That is precisely why the next generation of perioperative studies should prioritize early response biomarkers (imaging and blood-based) to enable adaptive decisions on surgical timing versus selective extension of neoadjuvant therapy.

### 3.3 Escalation can deepen responses

Critics often point to the toxicity profiles seen in recurrent/metastatic (R/M) trials, such as EAGLE, KESTREL, and CheckMate 651, to argue that adding CTLA-4 inhibition to PD-1 blockade unacceptably augments toxicity (48–51). However, extrapolating toxicity from long-term palliative regimens to brief neoadjuvant induction requires caution. In the curative setting, the tolerance for severe adverse events is lower, as any toxicity that delays or precludes surgery compromises the curative intent, but the exposure profile is fundamentally different.

The biological rationale for neoadjuvant CTLA-4 inclusion remains compelling. Whereas PD-1 inhibitors act primarily on effector cells within the tumor microenvironment (TME), CTLA-4 blockade predominantly augments early T-cell priming and clonal expansion in lymphoid compartments including the TDLNs (13, 52). This mechanism is most potent when the primary tumor and nodal basin are intact. Multiple trials indeed demonstrated deeper pathologic responses with CTLA-4 + PD-(L)1 compared to PD-(L)1 monotherapy, albeit with a higher irAE signal (2, 6, 13).

Therefore, the challenge is not whether to use dual blockade, but how to calibrate it. The answer likely lies in regimen optimization. Lessons from melanoma (OpACIN-neo) suggest that dosing strategies such as “flipped-dose” ipilimumab can preserve the priming benefit while reducing toxicity (53). Evidence supports a ‘hit early, hit smart’ strategy: utilizing a limited course of optimized dual blockade for neoadjuvant priming to maximize clonal expansion, then de-escalating to monotherapy or surveillance. This approach balances the imperative to improve survival with the ethical necessity of preserving quality of life and surgical eligibility.

### 3.4 The surgical-immunologic paradox

The rationale for dual-agent priming discussed above carries an surgical consequence: the priming apparatus, specifically TDLNs, should remain intact during the induction window (12, 52, 54). This creates a tension between immunologic optimization and established surgical oncologic principles. These mechanistic arguments do not justify de-escalation of neck dissection outside trials: elective neck dissection improves survival in early oral cavity SCC (55). A potential compromise lies in Sentinel Node Biopsy (SNB). In cN0 OSCC, SNB is a validated staging technique that could theoretically preserve the bulk of the lymphatic basin for immune priming while maintaining oncologic safety (56).

### 3.5 The field still underreports outcomes that matter to surgeons and patients

A persistent weakness across neoadjuvant trials is the mismatch between reported endpoints and the reality of curative HNSCC care. EFS/DFS/OS are essential but they do not capture whether cure is achieved with acceptable function. Critical surgical benchmarks such as R0 resection rates, margin control, revision burden, reconstruction success, fistula rates, tracheostomy duration, feeding tube dependence and time-to-adjuvant therapy are often relegated to supplementary appendices or omitted entirely. Too many trials provide only generic surgical complication without standardized grading (e.g., Clavien–Dindo), without differentiating local versus systemic complications and without reporting the downstream consequence most relevant for cure: whether complications delayed or prevented postoperative radiotherapy ± cisplatin.

Even the limited reconstructive literature illustrates why this gap matters. A multi-institutional retrospective series suggested that preoperative immunotherapy may increase wound complications in flap reconstruction (57), while a larger matched–control study found no significant difference (58). Both can be true in different contexts but the current evidence is too heterogeneous to reassure surgeons responsible for the consequences in a pathway that must deliver adjuvant RT in time.

Furthermore, the absence of standardized Patient-Reported Outcomes (PROs) represents a major blind spot. In a disease where local therapy can be disfiguring and functionally devastating, toxicity measurement must include the patient’s lived experience. Most current trials fail to systematically collect or report longitudinal Quality of Life (QoL) data using validated instruments like the EORTC QLQ-C30, QLQ-H&N35, and MD Anderson Dysphagia Inventory (MDADI) (59). If perioperative immunotherapy is to scale beyond tertiary centers, future registries must elevate these metrics to co-primary endpoints. Without this, the field risks repeating a familiar mistake: declaring that perioperative ICI is feasible while failing to measure the perioperative harms that determine whether feasibility translates into net patient benefit.

### 3.6 The next generation of neoadjuvant studies

The window trial era established feasibility and biological rationale for neoadjuvant immunotherapy but left a substantial implementation gap (2, 4–6, 9). Next-generation studies must move beyond maximizing pathologic response and demonstrate that immune priming can be achieved without compromising curative timelines. Response-adaptive strategies are attractive, yet in HNSCC they should remain hypothesis-generating until validated by hard endpoints and safeguards against loss of operability (47). In a disease where local failure carries major functional and survival consequences, delayed or modified surgery must be justified by prospective evidence rather than extrapolation from melanoma or lung cancer data where the surgical stakes are different.

This gap is underscored by KEYNOTE-689, which demonstrated efficacy but did not address the operational complexity of integrating immunotherapy into time-critical, high-risk surgical pathways. Ideally, adoption into standard practice would have been accompanied by a mandated phase IV implementation study to confirm that safety, timing and feasibility observed in expert centers are reproducible in routine care.

In summary, neoadjuvant window studies taught the field that immunotherapy can be delivered before surgery without routinely preventing resection in carefully selected patients, that pathologic response occurs but is inconsistent with PD 1 monotherapy, that deeper responses are achievable with combination regimens at the cost of higher toxicity and complexity, and that the lymphatic basin is central to the biology of response. They also taught a more uncomfortable lesson: the perioperative immunotherapy literature still does not measure the outcomes surgeons need to judge whether a regimen is safe to implement at scale. In 2026, the most important “next trial” is not only the next drug combination, but the next generation of trials that treat surgical quality, reconstruction, adjuvant timing and long term function as required endpoints rather than afterthoughts. Key messages from this section are summarized in **Box 2**.

Box 2 **Key messages.**

- Window studies show feasibility when timelines are engineered, not automatically in routine practice.
- Monotherapy can induce pathologic response, but deep responses remain inconsistent and timing influences what pathology can detect.
- Next-generation studies should report surgical endpoints and PROs alongside efficacy.

## 4 The surgical perspective of perioperative safety

Perioperative safety in the PD-1 era should be defined by failures in the curative pathway, not by whether an individual complication can be attributed to immunotherapy. The clinically relevant endpoints are.

- loss of operability or surgical simplicity during the neoadjuvant window.
- perioperative morbidity that compromises reconstruction or delays postoperative radiotherapy (± cisplatin).
- irAEs that destabilize peri-anesthetic risk or derail adjuvant delivery.

### 4.1 Operability and surgical timing

Across trials, the early fear that neoadjuvant ICI toxicity would routinely make patients inoperable has largely been dispelled. Short-course regimens have generally proceeded to surgery without treatment-related delays. For surgeons, the primary safety concern is indeed protection of the curative window. Any interval event that leads to loss of resectability or forces abandonment of definitive surgery is an irreversible harm. This risk includes preoperative attrition from tumor progression, new medical contraindications (including clinically significant irAEs) and patient withdrawal. Crucially, perioperative feasibility should not be treated as a binary outcome of reaching the operating room. Even when resection remains possible, interval progression or treatment-related effects may necessitate more extensive surgery with greater functional morbidity, thereby reducing quality of life despite preserved operability. These surgeon-centered outcomes are not systematically captured in most neoadjuvant ICI trials and should be treated as plausible risks.

Current quantification is unfortunately binary and comes from KEYNOTE−689. Among the 361 patients who received neoadjuvant pembrolizumab, 11% (38/361) did not undergo surgery. Only 1.4% (5/361) were cancelled for adverse reactions, whereas disease progression (4%; 15/361) and patient decision (3%; 10/361) accounted for a larger share (1). Phase II experiences show a similar pattern in smaller, selected cohorts. Most patients reach surgery, but a minority do not, sometimes due to rapid progression. In the perioperative pembrolizumab study by Wise−Draper et al., four patients did not proceed to surgical resection, including two with rapid progression (43). In IMCISION, three patients did not undergo surgery, including one patient whose progressive disease precluded surgery, alongside cases where post-enrollment assessment revealed unresectability at baseline (6).

The duration of the neoadjuvant interval remains clinically important but the current evidence does not support a simple imperative that longer windows are unsafe. Some longer-window designs have reported 0 attrition in highly selected populations (e.g., CIAO with an 8−week surgical window) (5). These examples highlight that attrition can reflect both aggressive biology, staging and selection limitations, not simply the passage of time.

The goal is to protect the curative window. Neoadjuvant ICI should be integrated with a fixed surgical date, close clinical surveillance and clear escape criteria (proceed directly to surgery if progression is suspected). In borderline-resectable or biologically aggressive disease, immediate surgery or potentially a shorter window approach may be preferable, but the final decision should remain an MDT decision, individualized to tumor behavior and patient fitness.

### 4.2 The “sticky neck” problem

“Sticky neck” concerns in HNSCC are real for surgeons who have treated the post-(chemo)radiotherapy salvage phenotype patients with obliterated planes, friable microvasculature, nerve tethering and non-anatomic dissection. The critical question is whether neoadjuvant checkpoint blockade creates a comparable technical problem that increases bleeding, cranial nerve injury or reconstruction compromise. The evidence here is thin. Most neoadjuvant ICI trials in HNSCC report no surgical delays and global complication rates, but they rarely prespecify or quantify the operative-field endpoints that would actually validate or refute stickiness: operative time, blood loss, need for vessel sacrifice, rate of nerve deficits, unplanned changes in extent of neck dissection or surgeon-reported perspectives.

One of the few datasets is a small retrospective case-control analysis from Park et al. comparing surgery after neoadjuvant immunotherapy (n=5) versus neoadjuvant chemotherapy (n=6) in HNSCC (40). In that series, the immunotherapy cohort did not show increased operative time, intraoperative bleeding or hospital stay. Importantly, the authors report that neck dissection was performed in all patients with no nerve sacrifice due to severe adhesions or fibrosis in the immunotherapy group. The same paper acknowledges the theoretical concern that immunotherapy-related stromal changes could complicate dissection, but its operative findings argue against a fibrotic phenotype after neoadjuvant ICI, at least within the limits of a small retrospective cohort. This is valuable surgeon-facing evidence but at the same time insufficient for reassurance at scale because sample size is small and operative endpoints were not standardized.

A second signal is pseudoprogression: the immune-mediated nodal enlargement, edema, necrosis and treatment effect can mimic progression or extranodal extension on imaging and at surgery. Trials and real-world series repeatedly show that radiographic change in short neoadjuvant windows can be discordant with pathology, including apparent progression with meaningful treatment effect, creating risk of overtreatment if imaging is read without immunotherapy context (2, 40). This is where discordance becomes a problem.

In HNSCC, we currently lack validated, cross-trial comparable measures of operative difficulty after neoadjuvant ICI. The field is forced to infer from indirect proxies (delay rates, blood loss when reported, complication composites) and to triangulate from small surgical series. It would be prudent and interesting to standardize these measurements in future trials. At minimum, future perioperative ICI trials and registries should prospectively capture operative time, blood loss, unplanned changes in planned resections/neck dissection extent, nerve injury rates, vessel sacrifice/grafting and reconstruction-specific intraoperative contingencies (e.g., anastomosis revisions, thrombosis).

### 4.3 Reconstruction and wound outcomes

Reconstructive surgery is where immunotherapy-specific wound healing difficulties would be most clinically consequential, because flap failure, fistula or major wound breakdown can directly delay adjuvant RT/CRT and thereby threaten cure. The challenge is that complication rates in major head and neck surgery are already high and are strongly influenced by patient- and treatment-related factors that are common in this population including advanced disease stage, tobacco and alcohol exposure, anemia, malnutrition or sarcopenia, etc.

The available evidence is heterogeneous. In a multi-institutional observational series, Mays et al. reported high overall complication rates following head and neck reconstruction and identified an association between preoperative immunotherapy exposure and increased major recipient-site complications, whereas postoperative-only immunotherapy did not demonstrate a similar signal (57). Donor-site complications were also more frequent in patients receiving preoperative immunotherapy, particularly among those treated for recurrent disease or distant metastases.

By contrast, a matched analysis in treatment-naïve oral cavity SCC treated with neoadjuvant pembrolizumab did not demonstrate higher odds of composite postoperative adverse events after adjustment, surgical complexity variables (e.g., free flap reconstruction, tracheostomy) were dominant predictors of complications rather than immunotherapy exposure (60). A larger case-matched study examining complications after surgery for primary HNSCC likewise found no statistically significant increase in complications attributable to preoperative ICIs after matching, with age and free flap surgery again emerging as independent risk factors (58).

There is no phase III-quality evidence demonstrating a reproducible, clinically large signal that neoadjuvant PD-1 blockade causes flap failure or wound complications in resectable HNSCC. However, reconstruction-focused retrospective series show mixed signals and occasionally suggest increased complications with preoperative exposure, which should not be dismissed because the consequence of such complications is pathway-critical.

### 4.4 Who should not receive perioperative PD-1

Patient selection for perioperative ICI is not identical to selection for palliative ICI. In the curative setting, the tolerance for avoidable toxicity and for pathway disruption is lower because the opportunity cost is loss of cure. Evidence supporting perioperative PD-1 blockade is derived from highly selected trial populations. In KEYNOTE-689 and earlier neoadjuvant programs, eligibility was restricted to patients with ECOG performance status 0–1, and excluded those with active autoimmune disease requiring systemic therapy, chronic immunosuppression or solid-organ transplantation (1).

As a result, several common real-world clinical scenarios remain unstudied, including patients with ECOG ≥2, frailty or limited physiologic reserve, severe malnutrition or sarcopenia, borderline resectable primaries in which any treatment delay risks loss of local control, bulky N3 disease with a high penalty for interval progression, and inflammatory or connective tissue disorders requiring immunosuppression. These are not rare exceptions in head and neck oncology but frequent clinical realities. Importantly, benefits observed in KEYNOTE-689 should therefore not be assumed to apply to these populations. Until prospective evidence becomes available, use of perioperative PD-1 blockade in such patients should remain conservative, explicitly trial-anchored and guided by multidisciplinary assessment.

### 4.5 Protection of the anti-tumor immunity and the microbiome

The perioperative period is not a drug−free interval in immunological terms. Surgical trauma triggers stress responses and a wound−healing cascade that can transiently suppress anti−tumor immunity and may interact with the ICI backbone (61–63). Surgical quality therefore extends beyond margins and reconstruction to minimizing potentially avoidable immunologic cost: meticulous technique, multimodal analgesia, rapid mobilization, and enhanced−recovery pathways that reduce prolonged inflammation and infection risk. Short courses of corticosteroids used for anesthetic prophylaxis or irAE management are sometimes unavoidable, but routine or prolonged perioperative steroid exposure should be limited to clear indications (64–66). Future protocols could also explore strategies such as perioperative beta-blockade (to blunt catecholamine surges) and COX-2 inhibition (to reduce prostaglandin-mediated immunosuppression), both of which have shown promise in other solid tumors (62, 66).

Microbiome effects are another practical variable. In R/M HNSCC, systemic antibiotics and proton−pump inhibitors administered around ICI exposure have been associated with inferior outcomes, plausibly through disruption of gut microbiome–dependent T−cell T-cell priming (31, 67–69). In the perioperative setting, this supports a conservative approach to prophylaxis and acid−suppression: use antibiotics when indicated, but avoid extended courses and unnecessary PPIs. Emerging multi−omic data also suggest that intratumoral bacterial burden and myeloid ecosystem states can modulate ICI response (29, 30), reinforcing the need for prospective perioperative registries that capture antibiotic, PPI and steroid exposure and postoperative infections.

### 4.6 Perioperative irAEs

The integration of ICIs into the neoadjuvant window introduces a distinct safety paradigm for the surgeon. Unlike the metastatic setting, where toxicity is managed over months as a cumulative variable, the perioperative period is defined by the risk of acute physiologic destabilization that masquerades as standard surgical or acute complications (37, 70). While the overall incidence of high-grade irAEs in neoadjuvant PD-1 monotherapy trials is relatively low, reported as approximately 10% in KEYNOTE-689, the phenotype of these events presents a diagnostic challenge (1). In the immediate perioperative window, immune-mediated pneumonitis can mimic aspiration pneumonia or acute respiratory distress syndrome, while immune-mediated colitis can mimic Clostridium difficile infection or postoperative ileus (65, 71–73). Consequently, the primary hazard for the MDT is not the frequency of these events, but the risk of attribution error, where a treatable autoimmune flare is mismanaged as sepsis or surgical failure.

This risk is most acute regarding cardiovascular and endocrine toxicities, which act as silent disruptors of anesthetic stability. Although immune-mediated myocarditis is rare, affecting fewer than 1% of patients, it carries a high fatality rate and, unlike many other irAEs, typically presents early after ICI initiation (74, 75). Data from cardio-oncology registries indicate that ICI-myocarditis is an idiosyncratic, early-onset phenomenon, with a median time to presentation of just 17 to 21 days. This places the peak risk window precisely during the typical neoadjuvant surgical interval. Because myocarditis often presents with non-specific troponin elevation, arrhythmia or hemodynamic collapse, it is easily misdiagnosed as a standard perioperative myocardial infarction, particularly in the HNSCC population with risk factors such as smoking history.

Similarly, ICIs can cause endocrine irAEs such as hypophysitis and adrenal insufficiency, which may present with nonspecific features including refractory hypotension, hyponatremia, nausea and fatigue that overlap with common perioperative complications (76). In the setting of surgical stress, failure to produce adequate cortisol can mimic septic or hypovolemic shock, and without timely recognition and stress-dose glucocorticoids, adrenal insufficiency can progress to adrenal crisis (77).

Current data therefore does not support washout intervals between the last dose and incision but rather a more rigorous physiologic screening. Pharmacokinetic data demonstrates that PD-1 receptor occupancy on T-cells remains above 70% for months following a single dose, rendering the concept of a drug-free surgical window biologically illusory (78). Instead, the most defensible recommendation is active screening for occult toxicity. Routine preoperative assessment should include thyroid stimulating hormone (TSH) and morning cortisol levels to rule out subclinical endocrinopathy, alongside a low threshold for cardiac evaluation (ECG/troponin) in patients with non-specific fatigue.

Finally, surgeons must be empowered to initiate corticosteroids immediately for unexplained perioperative instability. Severe irAEs generally require prompt high-dose corticosteroids as first-line therapy and delayed treatment may worsen outcomes. Available observational and meta-analytic data suggest that corticosteroids used to manage irAEs do not consistently impair checkpoint inhibitor efficacy (37, 79).

### 4.7 Enhanced recovery after surgery

Enhanced Recovery After Surgery (ERAS) fundamentals remain dominant (nutrition, anemia optimization, smoking cessation, prehabilitation, airway strategy), but perioperative ICI adds exposures worth capturing because they are common biologically plausible modifiers of immune status and unevenly distributed across patients (80, 81). Evidence in HNSCC is not yet strong enough to mandate major ERAS deviations, but it is strong enough to justify prospective documentation so that associations with toxicity, wound outcomes and efficacy can be tested rigorously in the perioperative setting rather than debated retrospectively (78).

### 4.8 When neoadjuvant or perioperative PD-1 fails

As perioperative PD-1 becomes standard, MDTs will increasingly manage recurrences in PD-1–exposed patients. An unresolved question is whether prior exposure alters the feasibility, morbidity, and sequencing of salvage therapy after perioperative failure. Contemporary perioperative trials were not designed to address this.

Three failure scenarios require different, explicitly preplanned responses. First, suspected progression during the neoadjuvant window is time-critical because loss of resectability is an irreversible harm (**Table 2**). In KEYNOTE-689, progression accounted for 4% of patients who did not reach surgery, and CheckMate 358 similarly observed progression-driven attrition despite a short planned window (1, 9). When progression is suspected clinically or radiographically in a way that it could jeopardizes resection, the most defensible action is rapid reassessment and, if operability is preserved, earlier conversion to surgery rather than completing the planned neoadjuvant course. Interpretation is challenging because immune-related changes may mimic progression, and without validated biomarkers distinguishing between the two remains unreliable. In this setting, the iRECIST rationale to distinguish true progression from pseudoprogression in the R/M setting cannot be safely transferred.

**Table 2:** Practical multidisciplinary team (MDT) responses when neoadjuvant or perioperative PD-1 therapy fails or is interrupted.

Clinical scenario	Most practical MDT response
Suspected progression during the neoadjuvant window	If resectability will be threatened by further progression, convert to surgery rather than waiting to complete the planned ICI course.
Minimal or absent pathologic response at surgery	Not a validated reason to stop evidence-based postoperative PD-1 off protocol. Use standard postoperative therapy.
Relapse after perioperative PD-1	Manage as PD-1-exposed recurrent/metastatic HNSCC: chemotherapy or EGFR-based salvage. ICI rechallenge is most plausible in delayed settings.
Severe irAE interrupting planned perioperative therapy	Prioritize stabilization and curative local therapy. Treat the irAE promptly and individualize further systemic exposure.

Second, minimal or absent pathologic response at surgery is not a validated indication to discontinue postoperative PD-1 therapy outside clinical trials. Pathologic regression is a late surrogate of immune activity, and short neoadjuvant windows may induce immune priming without measurable histologic clearance. KEYNOTE-689 was not response−adapted, and no prospective data define a non-responder threshold rendering postoperative PD-1 futile. At present, lack of response should inform trial escalation and intensified surveillance, not deviation from evidence-based postoperative therapy.

Third, relapse after completing perioperative PD-1 creates a challenging systemic sequencing problem. Evidence specific to perioperative failure is absent but contemporary R/M data support that both rechallenge and chemotherapy-based salvage can be clinically meaningful in selected patients. In a large US real-world cohort of R/M HNSCC, Sun et al. reported benefits with ICI rechallenge, particularly in patients with a “delayed” rechallenge after intervening therapy (82). As this cohort largely included patients who discontinued treatment after 1–2 years of disease control, these findings support a mechanism of acquired resistance rather than primary refractoriness. Consequently, rechallenge is likely most viable for patients with a prolonged treatment-free interval, rather than those progressing during or immediately following perioperative blockade.

Equally important, post-ICI salvage chemotherapy retains substantial activity. While a retrospective series initially suggested high response rates to taxane/cetuximab regimens (83), this has now been validated prospectively. The Phase III trial by Fayette et al. in ICI-pretreated patients demonstrated a clinically meaningful median OS of nearly 9 months with standard chemotherapy (84). These data confirm that prior PD-1 exposure does not compromise the efficacy of subsequent cytotoxics, supporting a rational role for chemotherapy, particularly EGFR-based regimens, as the standard of care for early post-perioperative failure. Key messages from this chapter are summarized in **Box 3**.

Box 3 **Key messages.**

- Perioperative safety should be judged by protection of operability, reconstruction and timely adjuvant therapy.
- Evidence for a sticky neck or wound complications is mixed. The immediate clinical concern is pathway failure rather than a single complication.
- Patient selection matters: frail patients, unstable autoimmune disease and airway-threatening tumors require extra caution.

## 5 Response assessment and response-adapted therapy

If perioperative immunotherapy is moving toward response−adapted care, response assessment becomes the critical step. Neoadjuvant window studies have moved pathological response from an exploratory endpoint to a deliverable that must be reproducible. Contemporary systematic evidence highlights persistent heterogeneity in pathological response definitions, limiting cross-trial interpretability (8, 78). In contrast, when response assessment is standardized, perioperative ICI can become a reproducible platform for evaluating new regimens, identifying non-responders early enough to salvage the curative pathway and prospectively test de-escalation. The recent ambition toward a pan−tumor framework is therefore an implementation prerequisite.

### 5.1 Pathologic response is the best we have so far

Pathology remains the most definitive response readout after neoadjuvant ICI because it is a direct measurement of efficacy and is available in essentially every head and neck cancer center. However, utility is limited by a lack of harmonization rather than access. In mucosal HNSCC, heterogeneity persists in both the compartment scored (primary only versus primary + nodes) and the scoring method (tumor bed RVT versus “percent non-viable/necrosis” versus biopsy-to-resection change metrics) (47, 78). The problem is magnified by anatomy with treatment-related changes occurring in tight planes. This is compounded by anatomical confounders where mucosal primaries often exhibit pre-existing ulceration while lymph nodes may show distinct immune-mediated clearance or pseudoprogression. Given that nodal status drives prognosis and adjuvant intensification decisions, collapsing primary and nodal response into a single composite score risks obscuring biologically meaningful discordance.

Early immunotherapy trials often borrowed chemotherapy-era endpoints such as pCR (0% RVT) and MPR (≤10% RVT). These endpoints are easy to communicate but biologically crude for ICI. In lung cancer, the depth of pathological response correlate with EFS across the full spectrum of %RVT. Data from the CheckMate 816 trial established that every 1% increase in RVT corresponds to a 0.017 increase in the hazard ratio for EFS, confirming that clinically meaningful risk gradients extend well beyond the traditional 0% and 10% thresholds (45). HNSCC is likely similar: it is implausible that 11% RVT and 90% RVT represent equivalent risk simply because both are “non−MPR” (85, 86).

The most practical solution is therefore to treat %RVT as a continuous variable (ideally recorded in reproducible increments, e.g., 10%) and to report the complementary tumor bed components such as necrosis and immune-mediated regression. This is the core of the updated pan−tumor Society for Immunotherapy of Cancer (SITC)/International Neoadjuvant Melanoma Consortium (INMC) guidelines, which harmonize specimen handling, scoring, and reporting across tumor types and recommends continuous %RVT reporting in both primary tumors and lymph nodes, with optional categorical cutoffs (pCR = 0% RVT; MPR ≤10% RVT) (87). The guideline does not endorse a universal 50% RVT threshold to define “(non−)response”. An accompanying editorial underscores that for immunotherapy, pathologic response is better captured as a spectrum than as a binary label, and harmonized reporting is required if pathologic response is to become a credible intermediate endpoint for drug development (88).

### 5.2 Current neoadjuvant HNSCC trials approaches

The neoadjuvant HNSCC literature has used at least three distinct approaches, which should not be treated as equivalent.

First, immune related RVT methods: some programs have applied tumor bed–based RVT scoring frameworks that define pCR as 0% RVT and MPR as ≤10% RVT. A concrete HNSCC example is CheckMate 358 (neoadjuvant nivolumab), including also partial pathologic response as >10–50% RVT and assessed RVT across the resected tumor specimen including sampled lymph nodes (9). This approach aligns conceptually with pan tumor efforts to quantify the residual viable tumor fraction within the tumor bed as a continuous variable (87). It also aligns to response−adapted trial logic because the variable being measured is what adjuvant therapy is trying to eradicate: residual viable cancer.

Second, percent non-viable or necrosis dominant response systems. In the randomized phase II oral cavity trial by Schoenfeld et al., pathologic tumor response was graded by the percentage of treatment response (non-viable tumor) in the primary lesion (<10%, 10–<50%, ≥50%) using histologic features such as inflammatory infiltrates, necrosis, stromal scarring/fibrosis and granulomatous reaction (2). This approach is practical and captures immune mediated regression patterns, but it is not numerically equivalent to RVT tumor bed methods and it commonly focuses on the primary tumor rather than integrating nodal response.

Third, change from baseline biopsy approaches: some neoadjuvant programs quantified response as reduction in viable tumor cell percentage from baseline biopsy to on treatment surgical specimen, defining major response as 90–100% reduction (6, 89). This can be intrinsically vulnerable to baseline sampling error because a diagnostic biopsy is a random sample of a heterogeneous tumor. It is therefore difficult to compare this metric directly with RVT in tumor bed methods or to export it into multicenter phase III workflows unless baseline sampling is unusually standardized.

Hence, “pCR” and “MPR” are not self-evident terms in HNSCC. Any paper that quotes a pCR/MPR rate without clearly defining the denominator (primary only vs primary + nodes), the method (RVT vs necrosis dominant vs change from baseline), and the sampling strategy is not interpretable across trials.

### 5.3 What should be scored

If perioperative immunotherapy is going to support response−adapted therapy in HNSCC, the pathology dataset must explicitly separate the primary tumor bed and the nodal compartments, and it must be reported in a way that is reproducible.

A practical minimum dataset for neoadjuvant ICI trials and real world perioperative registries should include all of the following:

1. Regimen and timing must be described including agent(s), dose, dosing interval, number of cycles. Immune mediated regression is time dependent and biopsies at different time points invites false conclusions about regimen potency (2, 4–6, 9).
2. Primary tumor bed response should be reported as a continuous estimate of %RVT when feasible, and preferably in reproducible categories derived from that continuous estimate (e.g., levels per 10%), accompanied by reporting of necrosis and immune-mediated regression (87).
3. Nodal compartments should be reported per level, number of nodes per level, number of positive nodes per level and %RVT or response category in each involved level (46, 90).
4. Compartment inclusion must be explicit. Pathology report should state whether pathologic response assessment included primary, nodes or both. Without this, pCR/MPR rates cannot be compared meaningfully across trials. KEYNOTE 689, for example, used a stringent definition incorporating both primary tumor and nodal compartments for its pathological endpoints.

### 5.4 Defining the tumor bed for pathologic response assessment

For pathologic response assessment after neoadjuvant immunotherapy in HNSCC, the key metric is the percentage of residual viable tumor (%RVT) within the tumor bed. This requires a clear operational definition of the tumor bed itself. Unlike historic chemotherapy response scoring, which often emphasized necrosis, immune-mediated tumor regression typically produces a resorption phenotype in which viable tumor is replaced by fibrosis, neovascularization, and dense immune infiltrates rather than by necrosis alone (91).

For this reason, RVT should be assessed relative to the entire tumor bed, defined as the sum of remaining viable tumor, necrotic areas and stromal regression scar. In practical terms, this means that %RVT reflects how much viable tumor remains within the full footprint of the treated lesion, rather than how cellular a single microscopic field appears. Approaches that estimate tumor cellularity from selected high-power fields or fail to account for the regression scar will systematically overestimate residual tumor and may incorrectly classify patients as non-responders or exclude them from a major pathologic response category (92).

#### 5.4.1 Total tumor bed = RVT + necrosis + stromal regression

Even when digital tools are used, the critical step is not the choice of software but agreement on what is being measured and how the specimen is sampled. Visual estimation by experienced pathologists will remain the practical standard in many centers. Digital pathology platforms, such as QuPath, can support annotation, area quantification and auditability, but digital quantification should be reported as a method rather than assumed to be intrinsically superior (93). Translational programs such as DUTRELASCO illustrate the value of intensive tissue analysis, but also highlight that highly specialized assays require standardized specimen handling and are not universally available (13).

Two avoidable sources of inter-trial variability deserve emphasis. The first is inadequate sampling of the tumor bed. After neoadjuvant immunotherapy, immune-mediated regression, ulceration and fibrosis can obscure tumor boundaries, making random or limited sampling insufficient. Extrapolating from neoadjuvant melanoma protocols, reliable identification of major or complete pathologic response requires a dedicated neoadjuvant grossing approach in which the entire tumor bed, or at least a fully mapped cross-sectional representation in larger specimens, is submitted for histologic evaluation (44). Importantly, if the neoadjuvant context is not communicated to the pathologist at the time of grossing, the opportunity for accurate response assessment may be irreversibly lost.

The second source of variability relates to discordant responses between the primary tumor and regional lymph nodes. Neoadjuvant immunotherapy studies consistently report substantial heterogeneity between compartments, with discordance observed in up to 30–40% of cases (5, 6, 94). Collapsing primary and nodal responses into a single composite score risks obscuring biologically and clinically relevant differences. HNSCC allows precise nodal mapping and level-specific labeling, hence RVT in the primary tumor and in each nodal compartment should be reported separately to better reflect response biology and to inform postoperative risk stratification and adjuvant decision-making.

### 5.5 On-treatment imaging

Imaging remains the gatekeeper for the surgical window, yet immunotherapy introduces distinct biological confounders that challenge standard interpretation. While RECIST 1.1 relies on dimensional regression, neoadjuvant checkpoint blockade often induces an initial inflammatory response that stabilizes or increases tumor volume (95). This phenomenon of pseudoprogression prompted the development of iRECIST. For surgical decision-making, the critical issue is distinguishing true progression from pseudoprogression rather than categorizing conventional response states.

FDG−PET has the strong perioperative ICI−specific evidence as an early discriminator of pathologic response in HNSCC, largely driven by the IMCISION trial (90). In that cohort, early decreases in primary-tumor metabolic tumor volume and total lesion glycolysis identified pathological responders with high accuracy and continuous metabolic measures appeared more informative than categorical response criteria. Conversely, cervical lymph nodes exhibited paradoxical metabolic flare during ICI exposure, with increased size or SUVmax despite the absence of viable tumor. This underscores the need for compartment-specific interpretation and prespecified imaging timing.

MRI, particularly diffusion-weighted imaging and quantitative Apparent Diffusion Coefficient (ADC)-based analyses, is attractive as a response biomarker that avoids tracer logistics. Quantitative Diffusion-Weighted Imaging (DWI) features including ADC histogram and shape metrics have shown association with pathologic response, supporting feasibility but external validation and standardization of acquisition remain necessary (96). More recent texture-based MRI studies in HNSCC including deep learning enhanced radiomics have reported predictive performance in neoadjuvant chemoimmunotherapy settings (89, 97, 98). These findings are promising but are not yet directly generalizable to ICI-only pathways. In pure immunotherapy, the influx of immune effector cells can maintain high tissue cellularity (low ADC) despite tumor cell death, creating a pseudoprogression signal that confounds radiomic classifiers trained on cytotoxic shrinkage.

Artificial intelligence (AI) is increasingly explored for CT, MRI and PET in HNSCC, with the goal of extracting quantitative features linked to tumor biology and treatment response (99). In perioperative ICI pathways, AI could plausibly improve discrimination of immune flare versus true progression and enable multimodal response signatures integrating imaging with pathology and blood biomarkers. At present, neither MRI nor PET nor AI supports de-escalation of standard local therapy outside prospective trials.

### 5.6 Blood-based biomarkers

Circulating tumor DNA (ctDNA) is a plausible candidate to move perioperative ICI in HNSCC towards risk adaptive treatment because it can, in principle, measure minimal residual disease (MRD) after surgery and during adjuvant therapy. In non−HPV locally advanced SCCHN, Honoré et al. found that postoperative ctDNA positivity was associated with inferior recurrence-free and overall survival (100). This is the type of signal needed to justify escalation trials, but it is not sufficient to justify adjuvant omission when ctDNA is negative.

For HPV associated disease, circulating tumor HPV DNA and tumor tissue modified viral (TTMV) HPV DNA assays are increasingly studied for surveillance and recurrence detection. A prospective biomarker guided surveillance study in HPV positive oropharyngeal cancer reported feasibility of integrating plasma TTMV HPV DNA into follow up pathways (101). Another study on early postoperative dynamics showed that ctHPVDNA clearance immediately after surgery was highly time-dependent and that very early postoperative positivity often reflects delayed clearance rather than persistent disease, underscoring the importance of standardized sampling intervals (102). Longer-term kinetic analyses have shown that persistent or suboptimal TTMV clearance is associated with inferior progression-free survival (103). To further assess blood-based biomarkers, serial blood collection at prespecified time points and prospective plasma banking should increasingly be considered in trial protocols for perioperative immunotherapy studies.

The field is progressively integrating sophisticated peripheral immunomonitoring, such as cytokine profiling and omics-based immune cell characterization, to better capture dynamic treatment responses (104). Beyond ctDNA, most tissue and blood biomarkers (such as spatial immune architecture, T cell receptor clonality and myeloid signatures) are hypothesis generating and mechanistically informative but they are not broadly available or suitable for routine decision making in HNSCC (13). In contrast, pathology and standard imaging are readily implementable at scale, while ctDNA is approaching clinical utility for risk stratification while high-dimensional immune profiling continues to rely on specialized research infrastructure.

### 5.7 Response−adapted therapy

Currently, CPS is the only biomarker with direct clinical utility in perioperative HNSCC, as it determines eligibility for pembrolizumab, whereas response-based biomarkers remain investigational. Response−adapted therapy in HNSCC should not be described as a near term routine practice change. The stakes are higher than in several other tumor types because locoregional recurrence often produces catastrophic functional morbidity and salvage options are limited. The right near term goal is to make response assessment sufficiently standardized that de-escalation questions become testable rather than philosophical.

Melanoma provides a mature proof-of-concept for response-directed management after neoadjuvant ICI. OpACIN and OpACIN−neo demonstrated that neoadjuvant ipilimumab plus nivolumab can induce high pathological response rates and that pathologic response is strongly associated with relapse outcomes (21, 53). Building on these data, the PRADO trial operationalized response-directed surgery and adjuvant therapy where pathologic nodal response guided the extent of surgery and the need for adjuvant systemic therapy (105) A comparable organ-preservation logic is now being explored in cutaneous squamous cell carcinoma. In the randomized phase II MATISSE trial (neoadjuvant nivolumab with or without ipilimumab), a subset of patients with MPR declined surgery and radiotherapy after ICI and achieved durable clinical complete remission (106). The authors explicitly propose PET-based selection for response-guided de-escalation in future trials. MATISSE−2 extends this concept by prospectively testing a response-guided “wait-and-monitor” approach in patients who would otherwise require morbid surgery, with surgery reserved for insufficient responders or those with subsequent progression (107).

For mucosal HNSCC, the message is not that surgery or radiotherapy can currently be omitted, but that robust, compartment-specific response assessment can create a reproducible platform to (i) identify non-responders early enough to salvage the curative pathway, (ii) test intensified postoperative strategies in the highest-risk groups, and (iii) prospectively define stringent deep-response states as candidates for controlled de-intensification in future trials. Key messages from this section are summarized in **Box 4**.

Box 4 **Key messages.**

- Continuous %RVT, with separate reporting of primary and nodal compartments, is the most practical current standard for pathologic response assessment.
- Imaging and blood-based biomarkers are promising, but they do not yet justify de-escalation of surgery or RT/CRT.
- In HNSCC, response−adapted de-escalation remains trial-only because the cost of locoregional failure is high.

## 6 Discussion for next trials

With phase III perioperative immunotherapy now established, the next generation of trials will be defined less by new drugs than by how perioperative immunotherapy is integrated into the surgical cure pathway. Escalation strategies must deepen response while preserving operability, reconstructive feasibility and timely postoperative RT/CRT.

### 6.1 Escalation: intensify priming, not just therapy burden

The strongest surgical rationale for escalation is to increase pCR rates and reduce systemic failure among PD-1 non-responders while preserving the deliverability of the perioperative pathway. Window studies taught two durable facts. First, PD-1 monotherapy produces biologic activity and measurable pathologic regression in a subset, but deep eradication is uncommon and heterogeneous across patients and anatomic compartments. Second, increasing response depth is possible, but operational costs (immune toxicity, coordination burden, risk of missing adjuvant RT start benchmarks) matter in HNSCC more than in tumor sites with low-morbidity salvage.

Among escalation strategies, PD-1 plus CTLA-4 remains the most mechanistically coherent perioperative priming-intensification approach, because CTLA-4 blockade acts predominantly in lymphoid compartments to broaden and amplify early T-cell priming, while PD-1 blockade sustains effector function in tumor and circulation (37). In resectable oral cavity SCC, the randomized phase II study by Schoenfeld et al. directly supports this biological premise in humans: nivolumab plus ipilimumab produced higher pathologic response signals than nivolumab alone, but at the predictable cost of increased clinically meaningful immune-related toxicity, which is exactly what threatens a surgical timeline if not governed tightly (2). The IMCISION program reinforced the same direction, reporting (near-)complete pathologic responses in a subset with nivolumab plus ipilimumab, again with the inherent trade-off between response depth and the perioperative risk of irAEs (6).

It is important to discuss what these trials do not yet prove. They do not establish that dual checkpoint blockade should be adopted perioperatively in HNSCC, and they do not define the safest CTLA-4 dose, number of cycles or minimum exposure window that preserves curative deliverability. They do, however, justify the next generation of trials: short-course, perioperatively optimized CTLA-4 schedules designed explicitly to preserve operability, reconstruction feasibility and the ability to start adjuvant RT/CRT on time, with prespecified stop criteria and surgical endpoints treated as core outcomes.

The operative constraint is toxicity. Head and neck cure pathways have low tolerance for grade ≥3 irAEs that destabilize anesthesia risk, prolong recovery, or delay postoperative RT/CRT. Here, melanoma provides the most useful methodologic precedent. In OpACIN-neo, schedule and dose optimization of neoadjuvant ipilimumab/nivolumab reduced severe toxicity while maintaining high response rates (21, 53). The PRADO strategy went further by operationalizing response-directed surgery and adjuvant therapy after neoadjuvant dual blockade, showing how pathologic response can become an actionable variable once definitions and safety boundaries are prospectively controlled (105). The more recent NEJM melanoma neoadjuvant dual-checkpoint data reinforce that response depth can be profound, but only when toxicity is managed through thoughtful scheduling and strict perioperative governance (108). These melanoma lessons are best viewed as trial-design discipline for CTLA-4 escalation in a surgically unforgiving disease, not as permission for de-escalation in HNSCC.

Chemo-immunotherapy is the other escalation direction likely to mature. In R/M disease, adding chemotherapy to PD-1 mitigates early progression risk and increases response rates, supporting the logic that cytoreduction can bridge the time needed for immune priming (14). In resectable disease, the hypothesis is that a chemo-IO backbone may increase the frequency and magnitude of pathologic regression before surgery and reduce early relapse. This biologic rationale is reinforced by the inherently high chemosensitivity of HNSCC: platinum-based induction regimens achieve high objective response rates in locally advanced disease, and early chemo-immunotherapy cohorts have reported ORRs approaching 80–95% with corresponding high rates of R0 resection and pathologic response (109). The principal risk of intensified induction regimens is that increased myelosuppression or toxicity could elevate perioperative complications or delay adjuvant therapy, potentially offsetting any net pathway benefit. Systematic reviews and society guidance already emphasize that perioperative adoption of intensified regimens must be judged on deliverability and safety (17, 110).

### 6.2 Combinations beyond CTLA−4

A large portion of the current pipeline aims to avoid CTLA−4−level autoimmune toxicity by targeting resistance axes in the tumor microenvironment, particularly myeloid suppression and stromal exclusion. Credible perioperative combination should target a defined resistance mechanism, produce measurable biologic activity in the neoadjuvant window and demonstrate that surgery and adjuvant RT/CRT timing remain intact.

Early window studies illustrate the types of strategies currently being explored but remain hypothesis-generating rather than practice-defining. For example, the SNOW study evaluated sitravatinib plus nivolumab in oral cavity cancer and demonstrated measurable immune modulation within the preoperative window (111). Similarly, the CIAO trial assessed durvalumab with or without tremelimumab in resectable HNSCC and highlighted that different PD-(L)1/CTLA-4 combinations may produce distinct biologic and clinical signals (5). These studies provide mechanistic insight but are not designed to establish perioperative standards. Broader reviews of emerging checkpoints (e.g., LAG−3, TIGIT and others) underscore the breadth of the next−generation pipeline, but they also highlight that most of these strategies remain investigational (112).

For HPV-associated disease, antigen−specific priming strategies remain one of the most logical biologic directions, but the evidence base relevant is still early. Peer−reviewed combination immunotherapy data in HPV-associated cancers reinforce the concept that HPV status is not merely prognostic but an essentially different disease with specific immunotherapy backbones and response mechanisms (113). Future perioperative immunotherapy trials should stratify by HPV biology, because HPV-driven disease is uniquely suited to mechanistic immunotherapy evaluation.

### 6.3 De−escalation in HNSCC

De−escalation is where a surgeon’s conservatism is not philosophical. Locoregional failure in HNSCC often produces severe functional loss, and salvage after RT/CRT is frequently morbid and sometimes not feasible. This is why response−adapted omission of adjuvant RT/CRT cannot be justified by deep response rhetoric alone.

The evidence base is not yet sufficient to support routine de−escalation. The systematic review by Mastrolonardo et al. found that pathologic response after neoadjuvant immunotherapy correlates with improved DFS, but it does not validate pathologic response as a surrogate for cure across heterogeneous regimens and adjuvant approaches (47). Society−level reviews similarly emphasize that perioperative immunotherapy should not be used as a rationale to omit established curative local therapy outside prospective protocols (78).

However, the optimization of adjuvant duration represents a safer immediate frontier. Current adjuvant PD-1 regimens (15 doses) were selected pragmatically rather than by dose-duration optimization. Shortening this duration is a reasonable strategy but requires a shift from calendar-based prescribing to biology-based prescribing. Future protocols will likely leverage MRD assays, specifically ctDNA, to distinguish patients who are molecularly cured at resection from those requiring extended maintenance. Until such tools are standardized, the cost of de-escalation failure remains too high to justify off-protocol omission.

### 6.4 Chronotherapy

Chronotherapy is not a surgical intervention, but it is operationally relevant to perioperative programs because infusion scheduling is tightly linked to preoperative windows. A study−level meta−analysis of retrospective datasets across advanced cancers reported an association between earlier time−of−day ICI infusion and improved survival outcomes, while emphasizing substantial susceptibility to confounding and the need for prospective validation (114). A similar observation has been reported in a cohort of 255 patients with R/M HNSCC (115). The evidence is sufficiently intriguing and low−risk that perioperative trials and registries should record infusion timing prospectively and, where feasible, test timing as a stratification factor or randomized implementation question.

## 7 Conclusion

In summary, the most important next trials in resectable HNSCC are not defined only by new drugs, but by trial designs that treat surgical deliverability, nodal biology and salvage consequences as primary variables. PD-1 plus CTLA−4 escalation is biologically coherent and supported by early clinical and translational signals in HNSCC, but it must be tested in perioperative−safe schedules with explicit stop rules and surgical endpoints. De−escalation should remain trial−only. Salvage evidence must be built prospectively because perioperative PD-1 exposure changes the real−world relapse population faster than trials can currently answer.
